# Multi-Omics Profiling Approach to Asthma: An Evolving Paradigm

**DOI:** 10.3390/jpm12010066

**Published:** 2022-01-07

**Authors:** Yadu Gautam, Elisabet Johansson, Tesfaye B. Mersha

**Affiliations:** Division of Asthma Research, Cincinnati Children’s Hospital Medical Center, Department of Pediatrics, University of Cincinnati College of Medicine, 3333 Burnet Avenue, Cincinnati, OH 45229-3039, USA; Yadu.Gautam@cchmc.org (Y.G.); Hanna.Johansson@cchmc.org (E.J.)

**Keywords:** asthma, genomics, transcriptomics, epigenomics, proteomics, exposomics, metabolomics, multi-omics, machine learning

## Abstract

Asthma is a complex multifactorial and heterogeneous respiratory disease. Although genetics is a strong risk factor of asthma, external and internal exposures and their interactions with genetic factors also play important roles in the pathophysiology of asthma. Over the past decades, the application of high-throughput omics approaches has emerged and been applied to the field of asthma research for screening biomarkers such as genes, transcript, proteins, and metabolites in an unbiased fashion. Leveraging large-scale studies representative of diverse population-based omics data and integrating with clinical data has led to better profiling of asthma risk. Yet, to date, no omic-driven endotypes have been translated into clinical practice and management of asthma. In this article, we provide an overview of the current status of omics studies of asthma, namely, genomics, transcriptomics, epigenomics, proteomics, exposomics, and metabolomics. The current development of the multi-omics integrations of asthma is also briefly discussed. Biomarker discovery following multi-omics profiling could be challenging but useful for better disease phenotyping and endotyping that can translate into advances in asthma management and clinical care, ultimately leading to successful precision medicine approaches.

## 1. Introduction

Asthma is a complex respiratory disease characterized by wheezing and shortness of breath due to airway inflammation and hyper-responsiveness. It is a highly heterogeneous allergic airway disease comprising different pheno- and endo-types, many of which remain to be fully characterized [1]. The phenotypical diversity is mirrored by equally complex etiologies, and a variety of genetic and environmental factors are believed to interact to increase the risk of asthma in children and adults. It has become evident that diagnosis, monitoring, and treatment of asthma require a personalized approach; in order to realize this goal, increased efforts are needed to characterize distinct phenotypes, elucidate underlying mechanisms, and identify associated biomarkers [2,3]. Although hypothesis-based approaches to asthma research have yielded important results, agnostic methodologies are well suited to handle the phenotypical complexity of asthma and have become increasingly important as tools for asthma biomarker discovery. Omics refer to unbiased approaches to the study of different classes of biological molecules, with genomics being the oldest and most established omics methodology. The pathogenesis of complex diseases such as asthma involves several cascades of events at various levels of omics including genomics of risk variants, transcriptomics of gene expression, epigenomics of gene regulation, proteomics, and metabolomics, which may have direct effects on disease etiology. A large number of genome-wide association studies (GWAS) have been conducted in the field of asthma over the last couple of decades, which has led to the identification of numerous candidate asthma risk genes, most notably the 17q12-21 locus which has been replicated in several GWAS studies [4,5] and which harbors a number of likely biologic candidate genes, including ORM1-like 3 (ORMDL3) and gasdermin B (GSDMB). Asthma-associated genetic loci, however, generally explain little of the disease risk, and genomics alone do not provide insights about the broader biological context in which the identified variants and associated genes operate. Other omics-based approaches have been used successfully to characterize the dynamic processes involved in asthma disease development and exacerbation. Transcriptomics, the systematic, unbiased characterization of RNA expression across the genome, has been used to profile distinct asthma endotypes, including T2-high versus T2-low endotypes [6,7]. Epigenomics, the genome-wide study of epigenetic changes, is a very important complement to genomics and can provide mechanistic links explaining interactions between genetic variations and environmental exposures. Proteomics and metabolomics to a large extent both rely on mass spectrometry (MS) to profile the asthma proteome and metabolome, respectively, and, while progress has been made in recent years especially in metabolomics, lack of standardization and the technical limitations of MS are challenges not yet fully overcome. The exposome refers to the totality of environmental exposures occurring to individuals over the course of their lives. Defining and measuring the entire exposome is a daunting challenge that has only begun to be addressed, but one aspect of the exposome, the microbiomes of the intestinal tract, the airways, and the skin, has proven to be crucially linked to the development of allergic disease including asthma, and the field of asthma microbiomics using culture-independent, unbiased methods has generated great interest over the last few decades.

In this review, we examine multiple omics approaches which are used to interrogate asthmatic patients to unravel key molecular signatures of biological processes such as transcription (transcriptome), translation (proteome), regulation of gene expression (epigenome), exposures (exposome and microbiome), their metabolites (metabolome), and their combined synergies (multi-omics). Several reviews on multi-omics of asthma have been previously reported. Kabesch and Tost reviewed recent findings in the genetics and epigenetics of asthma and allergy [8]. Hernandez-Pacheco et al. reviewed the genetics of asthma and treatment response [9]. Recently, Abdel-Aziz et al. reviewed reported major findings from different omics studies of asthma [10]. We aim to provide a comprehensive review on the current state of different omics of asthma. We also discuss the advantages and challenges of integrating individual omics into multi-omics approaches using systems biology and machine learning technologies.

## 2. Genomics of Asthma

Genomics is the characterization of an organism’s entire genetic content. Asthma has long been known to be heritable (genetic) as underscored by the fact that asthma runs in families, and offspring of asthmatic parents are at higher risk of developing asthma, with a higher rate of co-occurrence among monozygotic twins in comparison to dizygotic twins [11,12,13]. The heritability estimates were as large as 55–74% in adults [14,15] and up to 90% in children [16]. These population-based studies point to genetic risk factors for asthma predisposition. However, the heritability is polygenic in nature with multiple genetic variants contributing to the disease risk. In this section, we overview our current understanding of asthma susceptibility genes/loci, focusing on linkage, candidate gene, genome wide association, admixture analysis, and emerging high-throughput sequencing technologies on asthma.

### 2.1. Linkage and Candidate Gene Approach of Asthma

Family-based linkage, positional cloning, and subsequent candidate gene association analyses were the early approaches of mapping disease susceptibility genes related to asthma. The first linkage analysis on asthma linked immunoglobulin E (IgE) responses underlying asthma and atopy to chromosome 11q [17]. Linkage analyses usually identify a broader locus encoding several putative genes, which are further explored at a higher resolution through positional cloning and positional candidate genes to narrow down the linkage to a gene or a sequence of interest. Until 2006, linkage and positional cloning analysis identified eight genes of interest *ADAM33*, *DPP10*, *PHF11*, *NPSR1*, *HLA-G*, *CYFIP1*, and *OPN3* [18]. The candidate gene association analyses identified several asthma susceptibility genes including highly replicated genes *IL4*, *IL13*, *ADRB2, TNF*, *HLA-DRB1*, *HLA-DQB1*, *FCER1B*, *IL4RA*, *CD14*, and *ADAM33* [18,19,20]. However, linkage analysis is mainly conducted on trios data, limiting the accessibility of more samples, and it suffers from low power to detect risk variants with a small effect size [21]. Additionally, linkage analysis has limited genomic coverage and is biased toward the loci more likely to exhibit familial segregation, thus missing the unbiased detection of the population-based mutations associated with asthma. Candidate gene association studies provided better resolution than linkage analysis; however, it is mainly limited to refuting or ratifying genes selected with some knowledge-based approaches. Moreover, it is often performed on small samples and, thus, marred by low power and a high false positive rate [21]. As genotyping technology advanced to next-generation sequencing, and efficient computation tools such as PLINK [22] became readily available, the discovery of disease risk variants shifted from linkage studies to case–control genome-wide association studies (GWASs) for polygenic diseases including asthma.

### 2.2. Genome-Wide Association Studies (GWASs) in Asthma

The main strength of GWASs is their ability to systematically and unbiasedly explore novel variants associated with a disease [23]. Since 2007, GWASs have identified hundreds of genetic variants that potentially contribute to asthma and have provided a rich genome-wide atlas of disease susceptibility variants [24]. The first GWAS on asthma was based on 994 childhood onset asthma patients and 1243 non-asthmatic controls of European ancestry [25]. Over 317,000 SNPs were genotyped, and a novel risk locus on chromosome 17q12-21 was reported [25]. The locus 17q12-21 encodes several genes including *ORMDL3*, *GSDMB*, *ZBPB2*, and *IKZF2*, which in fact were linked to asthma in subsequent GWAS and eQTL analyses [4,5,26], and it is one of the most replicated asthma loci to date. The locus has been expanded to include flanking regions covering *PGAP3*, *ERBB2*, and *GSDMA* as other potential genes associated with asthma [27].

Over the last 14 years, several large-scale consortium-based GWASs and meta-analyses have improved our understanding of the genetic underpinning of asthma. Such analyses benefited from large sample sizes with improved power to detect the variants with small effects and provided better control of false positive discovery. The GABRIEL Consortium was the first large-scale meta-analysis on asthma which combined data from 23 different studies of European ancestry [5] and included 10,365 asthma cases and 16,110 controls. The Study of African Americans, Asthma, Genes, and Environments (SAGE II) project is a gene environmental interaction study of asthma in African Americans (AAs) with 812 asthma cases and 415 controls [28]. Similarly, the Genes Environments and Admixture in Latinos (GALA II) project (2022 asthma cases and 2135 controls) was focused on asthma and related clinical traits and environmental interactions in Latinos [29]. The EVE Consortium performed multi-ethnic meta-analysis of European ancestry, AAs, and Latinos using nine different studies from North America that included SAGE II and GALA II [30]. The findings included associations in or near the genes *IL1RL1*, *TSLP*, *HLA-DQ*, *IL33*, *SMAD3*, *GSDMA*/*GSDMB* (17q21), and *IL2RB*. The EVE study also identified a novel association in AAs at the *PYHIN1* gene which was not observed for EA and Latinos [30]. The GWAS of GALA II replicated the 17q12 locus among the Latino population, as did the Latino meta-analysis on the EVE study. The SAGE II study identified one significant locus in the *PTCHD3* gene and two suggestive loci *SEMA3E* and *INSR* in the AA population. However, the study did not replicate any of the previously known asthma loci. A subsequent follow-up study of the potential association from the EVE Consortium identified the *KLK3* gene and an intergenic locus on 13q21 in the EA cohort, but no additional signals were identified in the AAs and Latinos [31]. These studies found a different genetic architecture of asthma for different ethnicities. In particular, they showed the challenge of finding asthma risk loci specific to AAs.

The Trans-National Asthma Genetic Consortium (TAGC) reported a large-scale meta-analysis of 142,000 individuals (23,948 asthma cases, 118,538 controls) of European, African, Latino, and Japanese ancestry [32]. The study identified a total of 18 signals, five of them novel to asthma (5q31.3, 6p22.1, 6q15, 12q13.3, 17q21.33), two independent signals on the previously known asthma signals from Japanese studies (6p21.22, 10p14), and two signals that were reported for asthma and hay fever (8q21.13, 16p13.13), in addition to replicating nine previously known signals. However, all the signals were dominated by the European samples, and no significant signal was detected for ancestry specific meta-analysis of AAs, Latinos, or Japanese. The Consortium on Asthma among African Ancestry Populations (CAAPA; 7009 asthma cases, 7645 controls) reported a meta-analysis of multiple admixed populations with varying degrees of African ancestry [33]. The CAAPA study identified two loci, 8p23 (nearby genes *ARHGEF10* and *MYOM2*) and 8q24 (in the gene *TATDN1*), that may be specific to African ancestry. The CAAPA study replicated known asthma signals including 17q12-21, but a cross-evaluation of these signals in TAGC study showed that the associations were mostly driven by the European ancestry. In particular, when only the subset of AA data was used, the locus did not identify any significant association with 17q12-21. The AA-only meta-analysis of the eMERGE network identified the *PTGES* gene as the African-specific locus associated with asthma [34].

**Figure 1:**
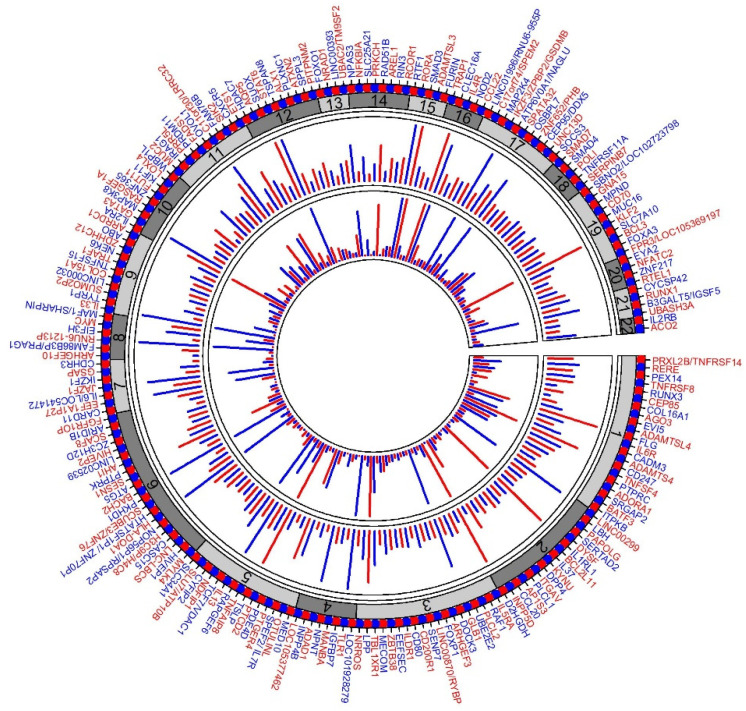
Circular plot of 212 asthma loci. Plot summarizing the 212 asthma loci described in Han et al. [35]. The first (outer) track shows the gene(s) representing the loci in alternating colors (red and blue). The second track with alternating shades of grays represents the chromosomes. The third track represents the association *p*-value from the UKBB + TAGC meta-analysis; each line represents −log10P of the strongest signal in the loci (for clarity, lines are truncated to ≤25). The innermost track represents the replication of each locus. Asthma GWAS loci with significance association *p*-value < 5 × 10^−8^ were accessed from the GWAS Catalog ((https://www.ebi.ac.uk/gwas/home, accessed on 10 December 2021), and the number of hits for each loci was counted. All replications of 17q12-21 were merged to a single locus. The lengths of the lines show the number of hits (for clarity, lines are truncated for lengths up to 20).

The most recent GWAS on asthma was conducted using UK Biobank (UKBB) data with 64,538 cases and 329,321 controls of European ancestry; subsequently a meta-analysis of the UKBB GWAS and the TAGC GWAS was performed [35]. To our knowledge, this is the largest GWAS study of asthma to date. The UKBB GWAS alone identified 145 significant loci, of which 41 loci were novel findings, and the remaining loci overlapped with loci previously associated with asthma or related phenotypes. The meta-analysis identified 167 significant loci, 58 of which were novel findings. Eight loci from the UKBB GWAS did not reach the significance threshold in the meta-analysis. In total, the study reported 66 novel asthma associations. Altogether, the authors reported 212 significant asthma susceptibility loci (Figure 1). Among the previously known 146 asthma loci, 109 loci were replicated at the GWAS significance level, whereas 34 loci were marginally replicated. Only three previously known asthma signals (8q23.3, 4q12, and 14q13.3) failed to replicate.

Two recent large-scale GWASs compared childhood-onset asthma (COA) vs. adult-onset asthma (AOA) [36,37] using the UKBB data on asthma. Both studies showed that childhood-onset asthma (COA) and adult-onset asthma (AOA) had partly diverse and partly shared genetic architectures with higher heritability observed for COA than AOA. There was moderate genetic correlation between the two cohorts (genetic correlation = 0.67) [37]. Both studies showed that the genetic risk factors for AOA were mostly a subset of COA with reduced heritability. Subgroup analysis based on age of onset in most of the large-scale GWAS studies has consistently shown that the most revered asthma locus in the GWAS era 17q12-21 is associated with COA but not AOA [5,36,37].

### 2.3. Admixture Mapping Analysis in Asthma

GWAS works best with homogeneous samples of single ancestral populations such as the European population. In admixed populations such as AAs or Latinos, differing ancestries may contribute to asthma susceptibility with varying frequency of risk variants. Multiple GWASs have shown that African-specific variants are not associated with asthma in European samples and vice versa. Thus, it is important to study the local ancestry variation and its association to asthma in the study of admixed populations. Admixture mapping is used to leverage the local ancestry variation among admixed samples to identify the ancestry risk variants [38]. The admixture mapping of Latinos in the GALA II study identified Native American ancestry at 6q21, centered on the gene *MUC22*, as significantly associated with decreased odds of asthma [39]. In the CAAPA study, the admixture mapping identified two genes *TCF21* and *TBPL1* in the locus 6q22.31–23.2 with increased African ancestry significantly associated with increased risk of asthma [33]. In an admixture mapping meta-analysis of childhood asthma in Latinos, a local ancestry region on locus 18q21 with Native American ancestry was found to be associated with childhood asthma [40]. Further fine mapping of the region localized the association to the *SMAD2* gene. Recent admixture mapping study of asthma in samples from the Canary Islanders showed that the North African ancestry in a locus 16q23.3 is associate with increased risk of asthma [41]. A fine mapping with the whole genome sequence localized the association at gene *PLCG2* as a novel risk locus of asthma.

### 2.4. Whole-Genome and Whole-Exome Sequencing in Asthma

Whole-genome sequencing (WGS) and whole-exome sequencing (WES) are emerging technologies in human genome study and could be useful to identify the disease susceptibility structural variants such as copy number variations and rare variants that GWASs do not capture. To our knowledge, few asthma studies have been performed using WGS, and WES is primarily used in rare variant discovery. Campbell et al. [42] performed a WGS on 16 samples from Hutterite families and identified 1960 CNVs, 19 nonsense or splice-site single nucleotide variants (SNVs), and 18 out-of-frame insertions or deletions associated with asthma. WES can be a cost-effective way to screen genetic variation compared to the WGS. An alternative to screening the whole genome for disease-causing variants is to limit the analysis to the exome, the protein-coding part of the genome. Although whole-exome sequencing (WES) has primarily been used for rare monogenic diseases, a few studies have applied WES to the search for genetic variants associated with asthma. Ten novel exonic variants were found to co-segregate with pediatric asthma in a single family [43]. Bogadi et al. [44] used WES to identify 21 exonic variants associated with pediatric asthma, and, of the identified variants, eight were novel. To translate the findings from WGS and WES to clinical implementation and personalized medicine in asthma, further studies with larger sample sizes and robust validation of the findings are required.

Moving asthma GWAS findings into clinical utility has several challenges including determination of the variant, the regulatory effect and associated tissue, the gene, the pathway, and the mechanism. A novel integrative genomics approach that combines GWAS information with gene expression and other multi-omics data for the discovery of potential clinically actionable biomarkers and identification of gene regulatory networks and biological pathways enriched for genetic variants is needed.

## 3. Transcriptomics Analysis in Asthma

The transcriptomic study of asthma involves profiling the expression of all types of RNA transcripts (e.g., messenger RNA (mRNA), noncoding RNA (ncRNA), microRNA (miRNA)) in a specific cell or tissue type. Two methods are primarily available for transcriptomic study: DNA microarrays and RNA-sequencing (RNA-Seq). Most of the transcriptomic studies of asthma have been conducted using DNA microarrays. RNA-Seq is an emerging technology with a promising outlook; however, it is costlier than the microarray. Sample groups mainly consist of asthmatic cases and healthy controls, and cases can be further divided into subgroups such as mild, moderate, and severe. Meta-analyses of multiple independent studies can improve the power to detect marginal signals and can improve the reproducibility of the results [45,46,47]. Gene set enrichment and network analysis on differentially expressed genes (DEGs) can be performed to identify the pathways and gene networks that the DEGs are most likely to affect. Differential gene expression analysis is used to identify the genes or transcripts that are DEGs between the sample groups utilizing one or more of the analytic tools available such as limma [48], edgeR [49], and DESeq2 [50]. NetworkAnalyst is a web tool that provided a comprehensive platform for differential gene expression analysis, meta-analysis, and network analysis [45].

Over the decades, multiple transcriptome analyses of asthma have been performed, discovering many genes and pathways relevant to asthma and providing many insights into asthma disease mechanisms [51]. As gene expression is known to be cell- and tissue- specific, the transcriptomic profiles vary across different tissues and cells (The GTEX Project [52]). Cell- or tissue-specific transcriptomes are especially important in understanding the roles played by specific cells in asthma pathogenesis and distinguishing different asthma subphenotypes [51,53,54]. Transcriptomic studies of asthma are mainly performed on three classes of cells: blood cells (such as whole blood, peripheral blood mononuclear cells, lymphoblastoid B cells), airway epithelial cells (nasal epithelial, bronchial brushing), and sputum. Bronchoalveolar lavage (BAL) represents the internal environment within the lower respiratory tract. However, only one genome-wide transcriptomic profiling of asthma has been performed on BAL, which identified enrichment of cAMP signaling components in the BAL cell among adult severe asthmatics [55]; this study is not discussed in detail in this review. The whole-genome transcriptomic study of asthma consisted of several hundreds to thousands of DEGs, which are often explored using pathway and network analyses to understand the functional mechanisms of the genes contributing to asthma pathophysiology. Below, we overview the current state of asthma transcriptomic analyses across different classes of tissue types and outline some of the different results in complex tissues.

### 3.1. Blood Cell Transcriptomics of Asthma

Blood cells are easy to access and can be obtained using less invasive techniques than more relevant specimens from airways; nevertheless, blood cells are known to carry the expression of 80% of genes encoded in the human genome and can act as sentinels of diseases [56]. A recent transcriptomic study on severe asthma revealed that the differentially expressed genes on bronchial epithelial cells and fibroblasts were also dysregulated in peripheral blood mononuclear cells (PBMCs) [57], further validating the use of blood cells as a surrogate of airway cells for the transcriptomic profiling of asthma. Studies of asthma using blood cells are primarily focused on molecular phenotyping, asthma control, and acute exacerbation. Differential gene expression analysis using white blood cells from therapy-resistant severe asthma, controlled asthma, and healthy samples identified significant upregulation of bitter taste transduction receptor (TAS2R) pathways in severe asthma [58]. Another study on white blood cells found that children with controlled asthma and those with severe asthma have distinct gene expression profiles, revealing decreased glucocorticoid receptor signaling and increased activity of the mitogen-activated protein kinase and Jun kinase cascades in patients with severe asthma [59]. Croteau-Chonka et al. reported that two related biologic processes related to activation by TREM-1 (triggering receptor expressed on myeloid cells 1) and lipopolysaccharide were the signature transcriptomic profile on whole blood in 1170 adult asthma with varying asthma control status [60]. A gene expression profiling of PBMCs using a cluster analysis on 133 asthmatic children revealed that Th1/Th17-mediated asthma was associated with high neutrophil count and poor treatment control [61]. Regarding asthma exacerbation, transcriptomic profiling of PBMCs was constructed from 118 asthmatic individuals by comparing the gene expression during exacerbation episodes and during stable periods [62]. Cluster analysis of the DEGs revealed two significant gene signatures associated with exacerbations; the gene signatures related to innate immunity pathways characterized one cluster, and gene signatures related to lymphocyte activation through antigen receptors and subsequent downstream events of adaptive immunity characterized the other cluster [62]. Severe and mild/moderate asthma exhibited similar and highly correlated differential expression profiles in blood cells; however, the effect was stronger among severe asthmatics [63]. Upregulation of chemotaxis, migration, and myeloid cell trafficking and downregulation of B-lymphocyte development, hematopoietic progenitor cells, and lymphoid organ hypoplasia were observed in patients with severe asthma [63]. Hachin et al. studied the blood transcriptome and found that genes *GPRC5A*, *SFN*, and *ABCA1* were upregulated while genes *SERPINE1*, *GPRC5A*, *SFN*, *ABCA1*, *MKI67*, and *RRM2* were downregulated in PBMCs from patients with severe uncontrolled asthma [64].

### 3.2. Airway Epithelial Cell Transcriptomics of Asthma

Airway epithelial cells are primary cell types involved in the onset of inflammatory, hyperresponsiveness, and remodeling changes in asthma manifestation and are directly involved in asthma manifestation. Several transcriptomic studies of asthma have been conducted on airway epithelial cells from different compartments such as nasal epithelia, sputum, bronchial brushing, and bronchoalveolar lavage fluid. The nasal brushing and sputum are less invasive than bronchial brushing and bronchoalveolar lavage fluid. The transcriptomic profile of CD3^+^ T cells derived from sputum and bronchoalveolar lavage fluid was distinct to that from the endobronchial brushing [65]. Weighted gene co-expression network analysis on adults revealed reduced expression of genes in networks linked to epithelial growth and repair and neuronal function on airway epithelial cells of severe asthma [66]. A distinct signature of immune cell- and epithelial cell-specific expression patterns was observed in a study of gene expression analysis of nasal epithelial in childhood atopic asthma of AA [67]. The study identified 11 differentially expressed genes contributed by immune-specific cells, including B cells (*HLA-DMB* and *HLA-DOA*), mast cells (*CPA3*, *CTSG*, *TPSAB1*, and *TPSD1*), natural killer cells (*KLRB1*), T cells (*CD3G*, *CD6*, and *TCRA*), and multiple immune cells (*CST1*) [67]. Differences may suggest the diverse role of tissues in asthma development and the pathological changes of asthma, which may result in different endotypes of asthma.

Hachim et al. identified total of 10 genes (*GPRC5A*, *SFN*, *ABCA1*, *KRT8*, *TOP2A*, *SERPINE1*, *ANLN*, *MKI67*, *NEK2*, and *RRM2*) related to cell cycle and proliferation to be disturbed in the severe asthmatic bronchial epithelium and fibroblasts [64]. However, their study revealed that gene regulation varied across different tissues and asthma subtypes. For instance, *SERPINE1* and *RRM2* were upregulated in severe asthmatic bronchial epithelium and fibroblasts, *SFN*, *ABCA1*, *TOP2A*, *SERPINE1*, *MKI67*, and *NEK2* were upregulated in asthmatic bronchial epithelium, and *GPRC5A* and *KRT8* were upregulated only in asthmatic bronchial fibroblasts. Genes *MKI76*, *RRM2*, and *TOP2A* were upregulated in Th2 high epithelium.

Tsai et al. performed a large-scale meta-analysis of gene expression from different airway epithelial cells, consisting of eight different studies; two were based on childhood asthma with nasal brushing, and six were studies of adult asthmatics using bronchial epithelial brushings [68]. The analysis identified 450 differentially expressed genes with a consistent expression pattern between nasal and airway epithelial cells; however, stronger expression was observed in the airway epithelial cells. The results showed consistent upregulation of Th2 biomarker genes (*POSTN*, *CLCA1*, and *SERPINB1*), genes positively associated with mucus production *(IL13*, *FOXA3,* and *MUC5AC*), and genes related to endoplasmic reticulum stress (*AGR2* and *ERN1*). Downregulated genes included *MUC5B*, *FOXA2*, and *XBP1*. Meta-pathway analysis showed that pathways related to mucin synthesis and post-translational modification of mucins, synthesis of prostaglandins, thromboxanes, and eicosatetraenoic acid derivatives were enriched for upregulated genes, whereas downregulated genes were enriched in pathways related to interferon gamma signaling, tryptophan metabolism, and Notch and Hedgehog signaling [68].

A cross-sectional study on the U-BIOPRED cohort showed that the expression profiles for adult-onset severe asthma were different from those for childhood-onset severe asthma on airway epithelial cells [69]. The study identified that gene signatures related to inflammatory pathways involving eosinophils, mast cells, and group 3 innate lymphoid cells were more enriched for adult-onset severe asthma whereas signatures associated with induced lung injury were less enriched in adult-onset asthma.

### 3.3. Sputum Transcriptomics of Asthma

Sputum transcriptomic analyses showed increased expression of several genes related to tumor necrosis factor-α (*TNF-α*) signaling in the sputum of asthma participants with neutrophilic airway inflammation [7,70]. A recent study found that baseline sputum TNF receptor 1 (*TNFR1*) and receptor 2 (*TNFR2*) were significantly increased in neutrophilic vs. non-neutrophilic asthma [71]. The study also found increased sputum *TNFR1* and *TNFR2* in severe asthma, correlated with poorer lung function and worse asthma control, while sputum and serum *TNFR2* was correlated with increased frequent exacerbation. Cluster analysis of the sputum transcriptome in elderly asthma identified that the oxidative phosphorylation gene set was significantly enriched on the cluster with low sputum eosinophil and less severe airway obstruction, whereas the epithelial–mesenchymal transition gene set was enriched in more severe asthma [72]. The transcriptome on blood and sputum found that increased sputum-soluble TNF receptor levels in neutrophilic asthma were highly correlated with a number of airway monocytes, suggesting the role of airway monocytes in dysregulation of the TNF pathway in neutrophilic asthma [73].

### 3.4. RNA Sequencing in Asthma

RNA-Seq is an emerging technology in the field, and several transcriptomic studies of asthma using RNA-Seq data have been reported. A recent whole-genome transcriptomic study with RNA-Seq technology on peripheral blood identified *PTGDR2* as a significant biomarker of adult asthma [74]. The study found significant upregulation of *PTGDR2* in the subgroups of allergic asthma, asthma with chronic rhinosinusitis with nasal polyposis (CRSwNP), aspirin-exacerbated respiratory disease, eosinophilic asthma, and severe persistent asthma compared with non-asthmatic non-atopic controls. A prospective, longitudinal case–control study was conducted using the RNA-Seq data from nasal lavage and blood to identify changes in gene transcription during cold-associated asthma exacerbations in children [75]. The authors found that, in both viral and nonviral exacerbation, epithelial-associated *SMAD3* signaling was upregulated and lymphocyte response pathways were downregulated early in exacerbation, followed by later upregulation of effector pathways including epidermal growth factor receptor signaling, extracellular matrix production, mucus hypersecretion, and eosinophil activation. For the virus-associated exacerbations, additional inflammatory cell pathways were identified, while squamous cell pathways were associated with nonviral exacerbation. Using the RNA-Seq transcriptome in airway smooth muscles from asthmatic and non-asthmatic subjects, Banerjee et al. constructed a gene network using the differentially expressed genes and transcription factors and conducted differential co-expression analysis [76]. Eighty-three genes were found to be upregulated including two SLC family genes (*SLC2A12*, *SLC7A11*), and 38 genes were downregulated including several tubulin family genes (*TUBA1B*, *TUBB6*, *TUBA1A*, *TUBA1C*). The study also identified multiple enriched pathways including the pathways involved with herpes simplex virus infection, Hippo and TGF-β signaling, adherens junctions, gap junctions, and ferroptosis.

### 3.5. Single-Cell RNA Sequencing (scRNA-Seq) in Asthma

The ability to resolve transcriptional profiles of the entire transcriptome to the individual cell level is one of the most exciting advances in gene expression analysis to date. Cell populations can be differentiated according to the expression of unique transcripts, and differences in cell populations and their transcription across disease states can be compared. Several recent studies started to take advantage of this level of resolution for asthma. Profiling PBMCs of severe asthmatic and healthy controls using scRNA-Seq followed by transcriptomic analysis identified that several proinflammatory genes such as *JAK1*, *NEAT1*, and *IL32* were highly expressed in CD4^+^ T cells, CD8^+^ T cells, NK cells, and B cells of severe asthmatics [77]. Additionally, cell-type-specific heterogeneity of the transcriptomic profile was also observed. For example, the chemerin chemokine-like receptor 1 (CMKLR1) was downregulated in the monocytes of severe asthmatics, whereas it was upregulated in natural killer cells [77]. Single-cell RNA-Seq analysis of the bronchial biopsy from six chronic childhood asthma cases and six healthy controls identified a significantly increased number of goblet cells and mucous ciliated cells [78]. The goblet cell transcriptional analysis identified the upregulation of several proinflammatory and remodeling genes *NOS2*, *CEACAM5*, and *CST1*. In the mucous ciliated cells, the ciliated genes *FOXJ1* and *PIFO*, as well as genes *MUC5AC* and *CEACAM5*, were found to be differentially expressed. Mast cells were mainly identified among the asthmatics, and significantly higher expression of genes *TPSB2*, *TPSAB1*, *PTGS2*, and *HPGDS* was observed in the cluster of mast cells [79]. Li et al. identified a significantly higher proportion of monocytes, CD8^+^ T cells, and macrophages in the bronchoalveolar lavage fluid of asthmatic patients, and several cytokines and intracellular transduction regulators were shown to be associated with asthma exacerbation across multiple cell lines [80]. Single-cell RNA-Seq is overwhelmingly used for profiling the discrete cell types among asthmatics, and the differential gene expression analysis on the cell types is limited to few sample samples due to complexity and cost of analysis, although larger studies will certainly pave the way for optimizing the data pipeline for analyses of scRNA-Seq in asthmatic samples.

## 4. Epigenomics of Asthma

Epigenomics addresses genome-wide characterization of reversible chemical modifications of DNA or DNA-associated proteins impacting gene expression and regulation. It describes heritable mechanisms regulating genomic activity that occur in response to the environmental exposures and, hence, may play role in environment-related asthma pathogenesis [81]. Epigenomic variations can regulate gene expression without any change in the DNA sequence, are cell-type and tissue specific, and are responsive to different environmental exposures [82]. Increasing evidence suggests that epigenomic modifications related to asthma are modulated by various environmental exposures, such as air pollution, smoking, and diet, occur in utero and early life, and may have lasting impact in later life [82,83]. Epigenetic marks including DNA methylation, histone modifications, and changes in various noncoding RNAs and enzymes involved in reading, writing, and erasing of such marks have been studied [81,84]. Epigenetic editing might be a promising target both to study the function of those epigenetic modifications and to be used as a promising therapy for diseases such as asthma [85,86]. Studies have shown that different classes of epigenomic modifications play significant roles in the pathogenies of childhood and adulthood asthma.

DNA methylation is a biological process that attaches methyl groups to the cytosine at position 5′ in cytosine–phosphate–guanine (CpG) sites catalyzed by a family of DNA methyltransferases [87]. DNA methylation is often associated with gene expression, which can promote or suppress the gene via hypomethylation or hypermethylation, respectively [67,87]. Histone modifications can regulate the gene expression and are also involved in DNA repair and replication, alternative splicing, and DNA condensation [88,89]. Many forms of histone modification exist; histone methylation and acetylation are the two most commonly involve in regulation of gene expression. Various classes of noncoding RNA (ncRNA) such as miRNAs and long ncRNA are known for regulation of the gene expression, gene stability, and defense against foreign genetic elements [90,91]. Relative to DNA methylation, studies of histone modification and noncoding RNA on asthma are less frequent, but emerging. Studies on these epigenomic variations may bring important insight into asthma development and pathogenesis. Alhamwe et al. [92] reviewed the role of histone modifications in different allergic conditions including asthma. Additionally, Svitich et al. [92] and Weidner et al. [93] reviewed the role of miRNAs in asthma and allergy. Recently, Wasti, Liu, and Xiang reviewed the role of different epigenetic classes including histone modifications and miRNA in asthma [94]. Over decades, the studies on epigenomics of asthma and other allergic disorders were primarily conducted using DNA methylation [81,82]; in this review, we focus on the association of DNA methylation and asthma.

Epigenome-wide association studies (EWAS) investigate associations between asthma and changes in DNA methylation pattern across the whole genome. Infinium MethylationEPIC Beadchip and Infinium Human Methylation 450 k Beadchip are two DNA methylation arrays used for global DNA methylation profiling, which cover 850 K and 450 K CPGs, respectively, across the whole genome. Several bioinformatics tools are available to perform EWAS [95,96,97]. Bisulfite pyrosequencing techniques are available for the study of DNA methylation at single CpG sites [98].

DNA methylation profiles are known to be cell- and tissue-specific [99]. Most DNA methylation studies of asthma are either conducted on blood cells or airways cells. One study reported the methylation of sputum DNA in asthmatic vs. non-asthmatic smokers and showed that hypermethylation of the *PCDH20* gene in sputum is associated with asthma [100]. However, the study was conducted on selected candidate genes, and no epigenome-wide methylation of sputum DNA has been reported.

### 4.1. DNA Methylation of Blood Cells in Asthma

Epigenomic variations including DNA methylation are known to be associated with asthma and atopy at birth and studying epigenomic variations at early life is critical in understanding asthma development at later ages [101]. Several large-scale EWASs investigated the potential effects of maternal exposure to environmental factors during pregnancy on DNA methylation of blood cells in newborn children. A large-scale EWAS meta-analysis of 13 cohorts from the Pregnancy and Childhood Epigenetics (PACE) consortium identified several differentially methylated CpG sites in asthma-related genes [102]. Gruzieva et al. showed that the maternal exposures to inhalable particulate matter during pregnancy is associated with DNA methylation in newborns at several epigenetic markers annotated to genes linked to asthma and lung functions, including *NOTCH4* and *FAM14* [103].

DNA methylation studies of childhood asthma identified several genes with established roles in asthma and atopy associated with DNA methylation. EWAS of childhood asthma using PMBCs from the participants in the Inner-City Asthma Consortium identified several asthma-related genes such as *IL13*, *IL4*, and *RUNX3* with differentially methylated regions (DMRs) [104]. DNA methylation in cord blood mononuclear cells from the participants of the Infant Immune Study identified *SMAD3* methylation as associated with asthmatic children whose mothers were also asthmatic [105]. A large-scale epigenome-wide meta-analysis identified hypomethylated whole-blood DNA CpG sites on genes involved in the activation of eosinophils and cytotoxic T cells in childhood asthma from the MeDALL consortium [106]. Asthma-associated CpG sites were discovered in children of age 8, with one site replicated in children of age 4 and no replication in infants at birth. Another blood EWAS meta-analysis of eight cohorts of newborns identified nine CpGs and 35 regions with implications for asthma development [107]. In the same study, a cross-sectional meta-analysis of nine cohorts identified 179 CpGs and 36 regions with differential methylation [107]. Both studies showed little overlap between newborn and childhood meta-EWAS, which could suggest postnatal epigenomic changes likely linked to asthma. Chen et al. compared the gene expression and DNA methylation from peripheral blood mononuclear cells (PBMCs) of 97 children with atopic asthma and 97 controls and identified 284 genes that were both differentially expressed and differentially methylated [108]. Network analysis of 130 critical genes (35 genes with hypermethylation and decreased expression and 95 genes with hypomethylation and increased expression) identified multiple enriched pathways including asthma-related cytokine-cytokine interaction pathways [108].

Several EWASs have investigated DNA methylation in adult asthma using blood cells. Adult eosinophilic, paucigranulocytic, and neutrophilic asthma partly shared differential methylation profiles in blood monocytes, with the most distinct profile identified for neutrophilic asthma [109]. Three pathway networks, purine metabolism, calcium signaling, and ECM receptor interaction, were found to be associated with eosinophilic asthma, while two pathway networks, neuroactive ligand–receptor interaction and ubiquitin mediated proteolysis, were found to be associated with paucigranulocytic asthma, and one network with SFRP1 as a key node, was identified in neutrophilic asthma. A recent large-scale EWAS on blood identified differentially methylated novel genes including multiple drug target genes, *PDE4B* and *PPARG* in atopic asthma, and *PDE4B* and *AZU1* in nonatopic asthma [110].

### 4.2. DNA Methylation of Airways Cells in Asthma

An EWAS using nasal brushings identified several asthma- and atopy-related genes such as *ALOX15*, *CAPN14*, *HNMT*, and *POSTN* associated with DMRs [67]. DMRs or probes were also found in genes related to extracellular matrix, immunity, cell adhesion, epigenomic regulation, and airflow obstruction [67]. In another study, nasal methylomes (CpG sites and regions) were found to be associated with atopic asthma, elevated IgE and FeNO, and bronchodilator responses [111]. Discovered DMRs and CpGs were annotated to genes reported to be associated with allergic asthma, Th2 activation, and eosinophilia (*EPX*, *IL4*, *IL13*), as well as to genes *ACOT7* and *SLC25A25* implicated in a blood EWAS of asthma and IgE [111]. Yan et al. identified 12 genes (*STARD3NL*, *SLC35F4*, *TSR3*, *CDC42SE2*, *KLHL25*, *PLCB1*, *BUD13*, *OR2B3*, *GALR1*, *TMEM196*, *TEAD4*, and *ANAPC13*) with methylated CpG sites associated with exposure to violence and chronic stress, and they were further linked to childhood atopic asthma in a nasal epithelial EWAS meta-analysis [112]. EWAS followed by pyrosequencing on airway epithelial cells showed that exposure to traffic-related air pollution potentially modulated *TET1* methylation among childhood asthmatics [113]. An EWAS of Puerto Rico children with atopic asthma identified differentially methylated genes relevant to epithelial barrier function, including *CDHR3* and *CDH26*, and airway epithelial integrity and immune regulation, including *FBXL7*, *NTRK1*, and *SLC9A3* [114].

DNA methylation varies across different cell types and may not be directly translated from one cell type into other cell types. Blood cells which consisted of circulating components of immune cells and the cells from the respiratory tract showed distinct patterns of DNA methylation among asthmatics [115]. Recently, Lin et al. compared the differential DNA methylation profiles from asthmatics from PBMCs, nasal epithelial cells, and airway epithelia cells [116]. The authors found that asthma-associated methylation markers from nasal and airway epithelial cells provided better asthma classification than that from the PBMCs, even with a larger set of markers. Using the genes annotated to the top 100 DNAm probes, the authors found distinct methylation profiles across the three cell types with little overlap; however, the tissues shared several enriched pathways. Interestingly, the authors also discovered that the DNAm levels of the *CDH6* gene and *RAPGEF3* gene might interact with each other to jointly predict the risk of asthma, which suggests the pivotal role of cell–cell junctions in the pathological changes of asthma.

### 4.3. Whole-Genome Bisulfite Sequencing in Asthma

Although epigenome-wide studies have primarily been conducted using microarrays, whole-genome bisulfite sequencing (WGBS) has emerged in recent years as an option for epigenome-wide analysis [117]. WGBS covers CpG sites at a higher density compared with microarrays, but higher costs and the reduced sequence complexity that results from bisulfite conversion remain limitations. So far, very few studies have used WGBS in the field of asthma. Trump et al. [118] used WGBS in a study of associations between maternal stress and persistent wheezing in children under the age of 5 and found genome-wide alterations in DNA-methylation mainly in enhancer elements. Enrichment analysis for KEGG pathways showed that DMRs in children were enriched in the canonical and calcium-dependent Wnt signaling pathways.

## 5. Metabolomics of Asthma

Metabolomics characterizes the small molecules (i.e., metabolites) present in a sample or matrix, including amino acids, fatty acids, carbohydrates, and other compounds in biofluids, cells, and tissues resulting from metabolic processes. Since the metabolome is often altered in response to physiological conditions, environmental exposures, disease processes, and medical treatments, metabolomics can be a very useful tool for endotyping and for the identification of biomarkers of disease and treatment response. Metabolites can be considered as the chemical language of interactions between different cells. The main technologies used for systematic analysis of metabolites are gas or liquid chromatography (GC or LC) coupled with mass spectrometry (MS). Nuclear magnetic resonance spectroscopy (NMR) is also used in metabolomic studies; however, since it has lower sensitivity than MS, it requires larger sample sizes.

Metabolomic studies can be either untargeted metabolic profiling or targeted studies of a single class of metabolites, both of which have been applied to the study of metabolites in asthma patients. Untargeted metabolomic profiling has been performed using MS on plasma, urine, stool, and exhaled breath samples from children with asthma [118,119,120,121,122]. In a study using untargeted LC–MS analysis of plasma metabolomes, children with severe asthma were distinguished by metabolic pathways associated with oxidative stress [121], in agreement with observations of increased oxidative stress in asthma exacerbations [123]. An association between plasma levels of ceramides and sphingomyelins at the age of 6 months and an increased risk of subsequent asthma development was found to be modified by 17q21 genotype [119].

The study of metabolomes in exhaled breath condensates is an area of extensive research in part because of the ease of sample collection, particularly suitable for collection from children. Exhaled breath metabolomics has been used to generate metabolite “fingerprints” of volatile organic compounds (VOCs) that could distinguish asthmatic children from healthy controls, as well as severe from non-severe asthma [124]. Exhaled VOCs have also been shown to differentiate children with and without recurrent wheeze [125], and a set of 17 VOCs in exhaled breath related to oxidative stress was shown to predict the subsequent development of asthma in preschool children with wheeze [126].

One limitation of untargeted metabolomics is that detection tends to be biased toward more abundant metabolites, thereby limiting the detection of important metabolites present in lower amounts, and targeted analyses of metabolites have a role in hypothesis-driven studies of the asthma metabolome. Results from observational studies and randomized trials have indicated that polyunsaturated fatty acids (PUFAs) promote immune system maturation and may protect against allergies [127]. Several targeted studies of PUFA plasma levels in children have shown that PUFAs in plasma are inversely associated with asthma, recurrent wheeze, and allergic sensitization [120,128,129]. Interestingly, in one of the studies, the association between PUFAs and asthma and/or wheeze was modified by umbilical cord blood 25-hydroxyvitamin D levels [120].

Although metabolomics holds promise for further discovery of asthma biomarkers and phenotype profiles, considerable methodological and analytical challenges remain. It is currently technically impossible to detect and characterize all metabolites in a biological specimen, and choice of sample preparation and analytical platform has a great impact on the results. Lack of standardization of analytical protocols makes comparisons of studies difficult and may be one factor behind failures to replicate findings. Moreover, the high degree of collinearity between many metabolites and the complex non-normal structures of metabolic profiles present difficulties for the statistical analysis of untargeted metabolomics, which may require adjustments of multiple-testing correction [130]. A range of unsupervised and supervised statistical methods and machine learning tools have been used in metabolomic statistical analysis [131], and further progress in this area can be expected to lead to improvements in the use of metabolomics for asthma phenotyping and biomarker discovery.

## 6. Proteomics of Asthma

Proteomics, the study of expressed proteins, is used to characterize protein identity, abundance, post-translational modifications, and/or interactions (or peptides). Protein levels cannot always be accurately predicted from mRNA levels [132], and proteomics has the potential to give a more precise and accurate picture of the physiological state at a given time during disease development. Proteomics aims to characterize, as closely as possible, the entire set of proteins and their isoforms in a cell, tissue, or biofluid. In practice, current technological limitations make it challenging to realize this goal, and proteomics studies are often focused on a subset of relevant proteins. An array of methods is used for protein detection in human samples. For more targeted studies of specific sets of proteins, antibody-based methods are often used, whereas MS, often coupled with liquid chromatography, gives an unbiased picture of the proteome. A recent development is the use of high-throughput protein microarrays that uses capture technology based on antibodies, aptamers, or antibody mimetics.

Compared with metabolomics, progress in the area of asthma proteomics has been slow, and early studies were mainly focused on targeted sets of cytokines and chemokines. In studies by the NHLBI Severe Asthma Research Program, bead-based multiplex immunoassays of 25 cytokines in bronchoalveolar lavage fluid were used to identify cytokine profiles associated with asthma phenotypes based on severity, predominant inflammatory cell type, and airway hyperresponsiveness [133,134]. Similarly, using immunoassays of 75 cytokines, chemokines, and growth factors in sputum, patterns of increased inflammatory factors were found in patients with severe asthma characterized by increases in neutrophils [135]. A targeted immunoassay of selected cytokines and chemokines in plasma was used to identify chemokines that predicted progression from wheeze at age 3 to asthma at age 6 [136]. Other studies have included arrays for more extensive sets of proteins in asthmatic individuals. Using an aptamer-based array comprising 1129 analytes, Lefaudeux et al. identified candidate biomarkers for four asthma phenotypes on the basis of severity and exacerbations, among other parameters. The candidate biomarkers included cytokines and proteins involved in cell adhesion, migration, and the extracellular matrix [137].

In recent years, studies using unbiased MS to characterize the asthma proteome have appeared. Schofield et al. used LC–MS analysis of sputum proteomes to identify proteomic clusters that were highly eosinophilic, highly neutrophilic, or highly atopic with low granulocytic inflammation. Candidate protein biomarkers for the phenotypes were identified [138]. In a study using shotgun MS, 18 proteins were identified that differed in abundance between allergic and nonallergic asthma, allergic rhinitis, and healthy controls [139].

As is the case for metabolomics, there is a lack of standardization of proteomics protocols that may contribute to variability between studies, and no current technology is able to identify all components of the human proteome. Studies to validate and replicate candidate protein biomarkers are needed in order to meet clinical needs.

## 7. Exposomics of Asthma

Exposomics is the study of environmental exposures (i.e., the exposome) and their effects on health and disease. Exposures may include aspects of the natural (e.g., air, water, and soil quality) and built environments (e.g., quality of workplace and housing, and access to fresh produce), as well as other chemical exposures and/or pollutants. These factors may exert biological responses, including inflammation, proinflammatory cytokine secretion, methylation, and gene expression changes, as well as increased responsiveness to cortisol. These responses can result in modifications to the epigenome, gene expression, and microbiome, and they could be linked with functional changes associated with diseases including asthma [140,141]. Asthma is a prototype of complex traits with both genetic and environmental causes. Environmental factors implicated in asthma risk include air pollution, smoking, environmental microbiotas, allergens, diet and nutrition [142,143,144,145]. Solid evidence exists for the association between childhood asthma risk and exposure to secondhand smoke (SHS) both pre- and postnatally [146,147]. Moreover, SHS has been found to interact with genetic factors to increase asthma risk [148,149,150]. Exposures to environmental microbial communities are believed to have a profound influence on the developing immune system. Evidence that children growing up on farms have a lower risk of asthma and other allergies was among the observations that led to the formulation of the so-called hygiene hypothesis, which stipulates that early-life exposures to diverse “friendly” microbiotas help protect against allergic disease by training the immune system to respond appropriately [151].

In addition to interactions with genetic factors, many exposures are likely to act synergistically to increase the risk of negative health outcomes over time. In order to better understand the health impact of environmental exposures and the interplay between these exposures and genetic factors, what is ideally needed is a systematic and standardized mapping of the total amount of exposures that an individual encounters from conception to death. This has given rise to the concept of the exposome. The exposome has been suggested to be composed of three domains: the general external, for example, climate and socioeconomic status, the specific external, which includes measurable exposures such as environmental pollution, radiation, allergens, microorganisms, diet, and medical interventions, and the internal domain, comprising internal biological factors such as oxidative stress, metabolic products, and organ-specific microbiota [152].

The three domains overlap and affect each other, such that variations of an internal exposure variable, for example, oxidative stress, may be determined by external exposome factors such as pollution, radiation, and diet. Moreover, the exposome is dynamic, and the effect of exposures may depend on age and critical time widows, which makes it challenging to estimate the total exposure to an environmental factor and its effect on the individual. Nevertheless, in recent years, new cohorts relevant to asthma and designed to explore the impact of the exposome have been initiated. Several recent cohort initiatives aimed at exploring the relationship between the exposome and allergic disease are currently ongoing. The European Human Early-Life Exposome Study (HELIX) is a large collaborative study based on six existing cohorts [153]. It was launched in 2014 with the aim of characterizing the early life exposome and its effect on children’s health outcomes, including asthma, and over 100 exposures have been evaluated. The Canadian Healthy Infant Longitudinal Development (CHILD) study is a longitudinal birth cohort study with over 3500 pregnant women who gave birth between 2009 and 2012 recruited from four provinces [154]. Biological, psychological, genetic, and environmental exposure data were collected in order to explore the developmental origins of allergy and asthma, as well as other chronic diseases. The Kingston Allergy Birth Cohort (KABC) is a smaller Canadian cohort designed to study the impact of prenatal and postnatal environmental exposures on the development of allergic disease [155]. Respiratory outcomes include wheezing up to 2 years of age but not asthma. The exposome-focused Environmental influences on Child Health Outcomes (ECHO) program is a 7 year North American initiative launched in 2016 that funds existing pediatric cohorts with a range of outcomes, including upper and lower airway health. One component of ECHO, The Children’s Respiratory and Environmental Workgroup (CREW), is a consortium of 12 birth cohorts from three scientific centers covering environmentally, racially, and ethnically diverse populations [156]. CREW aims to pool and harmonize existing data, as well as develop standardized protocols for the assessment of environmental exposures.

Multicohort initiatives can substantially increase statistical power, as well as participant diversity and ranges of exposure, but they require exposure assessment standardization, as well as harmonization of outcome definitions. Accurate and precise assessment of exposures remains challenging. A complement to the assessment of environmental exposures is the use of internal biomarkers, such as exhaled nitric oxide, DNA methylation, cotinine, oxidative stress markers, and others, that can serve as proxies of exposure [157]. Exposome Explorer [158] is a database of biomarkers of exposure to mostly pollution and dietary exposures containing information about biomarkers, their concentrations in various human biospecimens, and correlations with external exposure measurements. Markers of internal dose are often more relevant than external exposure assessments to the study of health effects, but they may be less useful for regulatory purposes since biomarkers of exposures are not necessarily specific. DNA methylation of certain genes, for example, can result from many different exposures, including different kinds of ambient air pollution and smoking.

Despite progress, exposome research is still at an early stage, and realizing the ambitious goal of characterizing the exposome and determining its role in the development of asthma is a considerable challenge. Many exposures relevant to asthma, for example, exposures to pets or air pollution, consist of very complex mixes of components, leading to confounding and correlations between exposures. Moreover, health outcomes are likely the result of multiple exposures that often act synergistically. Different statistical approaches have been applied to the analysis of exposome–outcome associations, including single-exposure regression, multiple-exposure regression-based methods, and various types of supervised clustering methods, all of which have their strengths and weaknesses [159,160]. Novel analytical strategies, as well as personalized and more precise methods for exposure assessments, can be expected to improve our understanding of the role the environment plays in asthma, and integration of genome, epigenome, and exposome data may yield important information about how genes and the environment interact to modify the risk of asthma and other allergic diseases.

## 8. Microbiomics of Asthma

### 8.1. The Human Microbiota

The human microbiota constitutes a very important part of the exposome and plays a crucial role in the maintenance of health. At the same time, the human microbiota interacts with the host and is affected by other aspects of the exposome, including diet, hygiene, air pollution, and the environmental microbiota [161,162]. It has long been known that germ-free mice without a microbiota have severely underdeveloped immune systems [163], and the human microbiome has been associated with a number of immune-related disorders, including asthma and allergy [164].

The gut microbiota is the largest and most diverse human microbiota, with the phyla Bacteroidetes, Firmicutes, and to a lesser extent Actinobacteria, Proteobacteria, Verrucomicrobia, and Fusobacteria, predominating in the normal gut microbiota [165]. A large body of literature on the relationship between the gut microbiota and disease has shown that the impact of the gut microbiota is in part systemic, and there is crosstalk across the so-called gut–lung axis [166]. Although much remains to be learned about the effect of the gut microbiota on the immune system, various bacterial species in the gut have been shown to modulate Th1/Th2 balance [167], induce regulatory T cells [168], and stimulate Th17 cell differentiation [169,170]. The microbial load in the lungs is low, and the lower airways were earlier believed to be sterile, but the emergence of culture-independent techniques has enabled systematic studies showing the presence of microbiota in both healthy and diseased lower airways [171]. Most of the bacteria in the lung belong to the Bacteroidetes, Firmicutes, and Proteobacteria [172], with considerable variation between individuals. Lastly, the importance of the skin microbiome for skin health has attracted increasing attention, and the association between *Staphylococci*, mainly *Staphylococcus aureus*, and atopic dermatitis is well established. The skin microbiome is implicated in the skin barrier and innate immune function [173,174,175]. Given the role of the skin barrier in cutaneous sensitization [176,177] and the fact that sensitization is a major risk factor for asthma [178,179], it can be expected that the skin microbiome is associated with asthma development. So far, however, reports on direct associations between asthma and the skin microbiome are lacking.

### 8.2. Technological Advances in Microbiome Research

In recent decades, culture-independent sequencing techniques have vastly expanded our knowledge of the human microbiome, its composition, and its role in health and disease. A number of studies have demonstrated important differences in microbiome composition between asthmatic individuals and healthy controls. Until recently, the predominant approach to culture-independent investigations of microbiotas has been the targeted sequencing of conserved genes containing hypervariable regions that can be used for taxonomic determination. For bacteria, the most commonly targeted gene is the 16S rRNA gene, whereas the nuclear ribosomal DNA internal transcribed spacer (ITS) region is usually used to identify fungi. Although target gene sequencing is still much in use for the study of microbiomes because of its low cost, sensitivity, and relative simplicity, the low taxonomic resolution obtained from the short segments of gene amplicons is a limitation. This is less of an issue for fungal communities, but 16S rRNA sequencing of bacteria rarely allows identification at the species level and is, therefore, best suited for broad profiling of bacterial microbiomes, although high-throughput sequencing of the full-length 16S rRNA gene has recently been shown to provide improved taxonomic resolution [180].

Although 16S rRNA sequencing has been very useful for the characterization of complex microbial communities, decreasing cost and improved high-throughput sequencing technology in recent years have made metagenomics a powerful alternative for taxonomic characterization of human microbiomes. Metagenomic sequencing produces sequence information for the entire genomes of all organisms in a sample and, in addition to taxonomic characterization, also reveals information on functional potential. Whereas target gene sequencing rarely allows taxonomic identification of bacteria beyond genus level, metagenomics at adequate sequencing depth yields information that can result in taxonomic identification at the species and sometimes strain level.

### 8.3. The Airway Microbiomes and Asthma

The microbiotas of the upper and lower airways have been shown to be distinct from each other [171]. Although there is no consensus on which microbiota is the most relevant to the study of asthma development, asthma has been associated with both upper and lower airway microbiotas. Sampling from the upper airways is considerably easier to perform than the more invasive procedures required to obtain samples from the lower airways, and studies of the airway microbiotas in infants and young children have mainly made use of nasal samples. At the genus level, increases in the Proteobacteria genus *Moraxella* in the nasal compartment is a common finding in asthmatic children compared with healthy controls [181,182], and *Moraxella* has also been associated with asthma exacerbations and eosinophil activation [183,184]. Asthma exacerbations have also been associated with decreases in the Actinobacteria genus *Corynebacterium* [183,184].

16S rRNA sequencing has been used in several studies to compare the microbiome in the lower airways of asthmatic individuals with that of controls. As in the upper airways, Proteobacteria are consistently found to be increased in sputum and bronchial epithelial brushings from asthmatic patients [171,185,186], whereas Bacteroidetes in the lower airways are more abundant in healthy controls [171,185].

Proteobacteria in the lower airways have also been associated with asthma severity [185,187]. Zhang et al. [185] used 16S rRNA pyrosequencing to compare the microbiomes in sputum samples from patients with severe vs. non-severe asthma, as well as healthy controls, and they found significant differences among the three groups. Firmicutes were increased in severe asthmatics compared with non-severe asthmatics and healthy controls, whereas Proteobacteria were most common in non-severe asthmatics. Bacteroidetes and Fusobacteria were decreased in both severe and non-severe asthmatics. At the genus level, several *Streptococcus* spp. were associated with asthma severity.

The fungal component of the microbiome has been studied in asthma patients using target gene sequencing. In a study using 16S rRNA and ITS sequencing to characterize the microbiome in asthma patients, the alpha diversity of both the fungal and the bacterial microbiotas from endobronchial brush samples were lower in asthmatics compared with controls, also in addition to being lower in patients with eosinophilic airway inflammation vs. patients with neutrophilic/mixed inflammation [188].

Metagenomic shotgun sequencing has also been applied to the study of airway microbiomes associated with asthma. Turturice et al. [189] used unsupervised clustering to identify two distinct asthmatic phenotypes with different cytokine and chemokine profiles. Furthermore, metagenomic sequencing showed that the phenotype associated with greater airway obstruction was also characterized by a lower airway microbiome enriched in *Streptococcus pneumoniae* and *Actinomyces* spp. Huang et al. [190] studied the sputum microbiome in both untreated asthma patients and patients treated with inhaled corticosteroids using metagenomic high-throughput sequencing, and they found that the α diversity of both the bacteriome and the mycobiome was lower in untreated patients compared with healthy controls. Several taxa were identified as potential biomarkers, including *Streptococcus*, *Gemella*, and *Neisseria* spp., which were increased in the untreated asthma group.

### 8.4. The Gut Microbiome and Asthma

Most studies of the gut microbiome have made use of stool samples, which are easily accessible even from neonates, and several studies based on 16S rRNA sequencing have linked the composition of the gut microbiota in infancy with the risk of asthma in later childhood [191,192,193]. Although the studies were in part contradictory with regard to critical timepoints and the protective effect of the Firmicutes genus *Veillonella*, two studies that used asthma in later childhood found an association between asthma and lower overall microbial Shannon diversity in the gut [191,193]. Interestingly, in the study by Stokholm et al. [191], the association between a more immature gut microbial composition in infancy and later asthma was only significant among children born to asthmatic mothers, suggesting an interaction between the gut microbiota and genetic or epigenomic factors.

Metagenomics has also been used to explore associations between the gut microbiome and asthma. A metagenomic study of the gut microbiome in asthma patients found evidence of lower microbial richness and a different microbial composition in the gut of adult asthma patients compared with non-asthmatic controls [194]. Metagenomic analysis also yielded evidence for functional changes in the gut microbiome, notably with regard to production of butyrate, a short-chain fatty acid with a role in inflammatory immune cell responses and associated with asthma risk [195,196]. This study demonstrates the potential of metagenomics not only to characterize microbiota composition but also to elucidate functional aspects of the gut microbiome, which may help to further our understanding of the role played by the gut microbiota in the development of asthma.

## 9. Integrating Omics in Asthma

**Figure 2:**
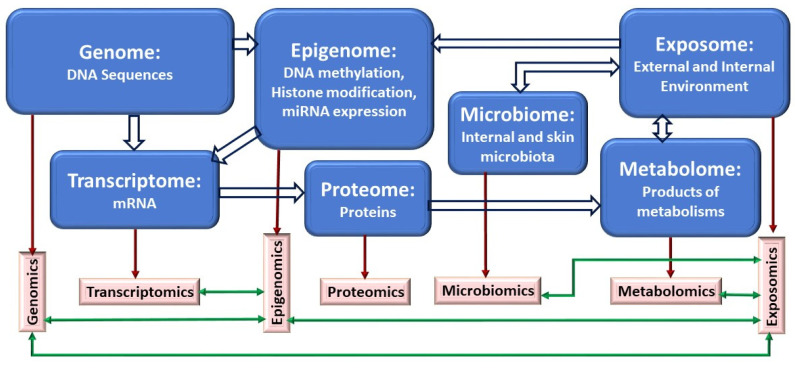
Schematic representation of relationships and interactions among types of biological molecules and their corresponding omics (top-down and bottom-up approaches). Block arrows indicate relationships between different classes of biological molecules, and green arrows represent potential interactions between omics datasets. One of the advantages of applying a multi-omics approach in biological systems is to understand the flow of information underlying disease and interpret the data in a holistic way in the context of biological networks and molecular interactions.

**Figure 3:**
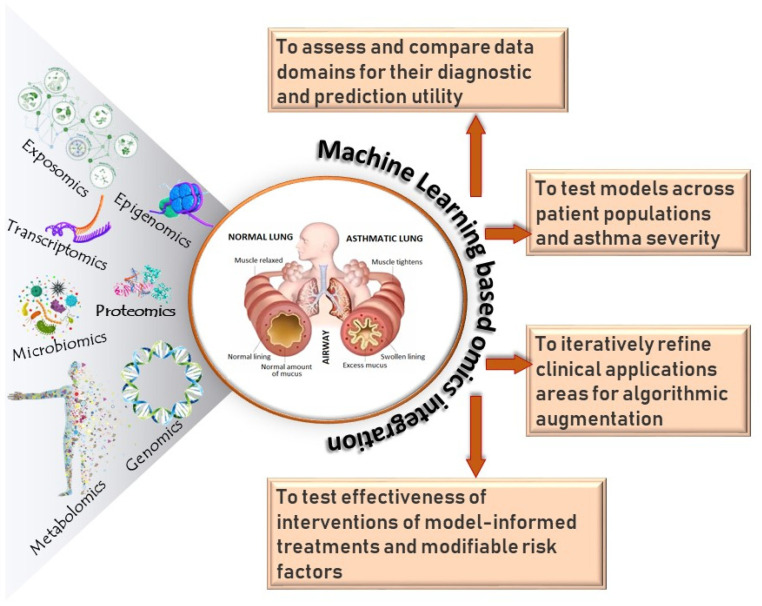
Overview of multi-omics integration in asthma. Mutli-omics integration could enhance our understanding of asthma including asthma development, asthma subtypes, and clinical symptoms, and it could lead to the development of novel intervention approaches and drug targets for asthma control and prevention.

While enormous progress has been made in understanding gene structure and regulation, translating molecular insights to clinical practice for the many asthmatic individuals has been challenging. Although single omics studies have contributed to a better understanding of the molecular profiles associated with asthma, they cannot fully capture the entire biological complexity of asthma. Multi-omics analysis holds great potential to molecularly characterize a wide array of complex diseases, including asthma [197,198]. Multi-omics analyses take advantage of genomics, transcriptomics, epigenomics, proteomics, metabolomics, the microbiome, and other omics areas to systematically understand health and disease states and uncover new biological insights into disease mechanisms. Applying multi-omics integration represents a holistic way to uncover relationships among biological molecules and their corresponding omics and phenotype (top-down and bottom-up approaches) and interpret the data in a context of biological networks and molecular interactions (Figure 2). Theoretically, multi-omics-based integration using machine learning has the potential to (a) assess and compare data domains for their diagnostic and prediction utility, (b) test models across patient populations and asthma severity, (c) iteratively refine clinical endotypes, and (d) test effectiveness of interventions of model-informed treatments and modifiable risk factors (Figure 3). However, despite continuous effort in integrating multi-omics data in asthma, truly integrated multi-omics analyses are in their infancy. A few notable attempts at multi-omics approaches in asthma research include the identification of biologically meaningful rhinovirus bronchiolitis endotypes [199], context-specific genetic mechanism [200], and clinically relevant biomarkers [201]. There are several reasons for multi-omics having so far generated few stable biomarkers for predicting asthma risk, including the lack of robust and reproducible omics data, whereas larger sample sizes and improved computational analysis are needed to powerfully integrate multi-omics data. Moreover, much of our understanding of genetic risk for asthma over the past decade came from studies of European ancestry. People of European ancestry account for about 16% of the world’s population, yet they represent 80% of participants in genetic studies of disease [202]. Due to the underlying genetic susceptibility and environmental exposure interactions, there is evidence that prevalence of immune phenotypes can vary by race [203]. There has been a lack of omics studies conducted among minority populations who bear a disproportionally high burden and incidence of asthma (almost twice as high in AAs (16%) compared to European Americans (8.2%) [204]. Additional efforts are needed to widen the diversity of multi-omics databases and develop analytical methods, including deep learning, to analyze, annotate, and integrate multi-omics data to inform precision medicine-based decision making [205]. New bioinformatic tools for data analysis are imperative given the large volume and complexity of available data toward the road of multi-omics application in precision medicine. Therefore, to achieve multi-omics integration and application to precision medicine asthma, it is important to address the current challenges by establishing a solid evidence base. This can be accomplished through more rigorous study designs, integration of high-dimensional data from various sources, development of computational approaches for large amounts of data, and a reduction in cost of omics analyses [206].

## 10. Conclusions and Future Directions

As we move forward, multi-ethnic cohorts with deep phenotyping, longitudinal phenotypes, better characterization of environmental determinants, and the application of new technologies and analytic approaches in “-omics” data integration are needed for better prediction, diagnosis, and biomarker development for treatment of asthma and related allergic diseases. These include next-generation sequencing (e.g., DNA-Seq, RNA-Seq (including scRNA-Seq)) and epigenomic approaches in appropriate tissues/cells along with advanced bioinformatic tools. Systematic integration of omics data (e.g., genomic, transcriptomic, epigenomic, proteomic, metabolomic, exposomic, microbiome), using multi-ethnic patient information from providers (e.g., electronic health records) and non-providers (e.g., smart phones, monitoring tools for environmental triggers) can, thus, provide valuable insights to resolve the clinical complexity and etiology of asthma. Precision medicine goes far beyond genetic sequence analysis, and integrating multi-omics data with deep phenotyping and clinical outcomes of the cohort offers a path to deeper functional insights into complex diseases such as asthma.

## Data Availability

Not applicable.
